# Cutaneous Squamous Cell Carcinoma Mimicking Kaposi Sarcoma in an HIV-Positive Patient: Diagnostic Challenges and Therapeutic Response to Liposomal Doxorubicin

**DOI:** 10.7759/cureus.94813

**Published:** 2025-10-17

**Authors:** Ana de Lourdes Torralbas Fitz, Sergio J Torralbas Fitz, Eliany Leon Figueredo, Elizabeth Blanco Espinosa, Idania Maria Cruzata Matos, Edurne Cárdenas Ferrer

**Affiliations:** 1 Oncology, Maputo Central Hospital, Maputo, MOZ; 2 Medicine, University of Miami Miller School of Medicine, Miami, USA; 3 General Medicine, Englewood Health Physician Network - Primary Care, New Jersey, USA; 4 General Practice, CEDA Orthopedic Group, Miami, USA; 5 Surgery, Hospital Arnaldo Milian, Santa Clara, CUB; 6 General Medicine, HCA Healthcare, Nevada, USA; 7 Pathology, Universidade Wutivi (UniTiva), Maputo, MOZ

**Keywords:** case report, chemotherapy, cutaneous squamous cell carcinoma, hiv, immunosuppression, kaposi sarcoma, liposomal doxorubicin

## Abstract

Cutaneous malignancies are among the most common cancers worldwide, with cutaneous squamous cell carcinoma (cSCC) being the second most frequent non-melanoma skin cancer. In people living with HIV (PLHIV), cSCC may present atypically and overlap clinically with opportunistic tumors such as Kaposi sarcoma (KS), complicating diagnosis and management. Immunosuppression increases the risk of aggressive and multifocal cSCC, often delaying diagnosis. Liposomal doxorubicin, an anthracycline-based chemotherapy standardly used for KS, has shown limited off-label activity against other cutaneous malignancies. This report describes an unusual case of HIV-associated cSCC that initially mimicked Kaposi sarcoma and responded unexpectedly to liposomal doxorubicin.

We report a 64-year-old woman with well-controlled HIV infection on antiretroviral therapy who presented with a progressively enlarging, exophytic scalp lesion. The initial punch biopsy was inconclusive. Imaging showed no bony involvement, although advanced modalities (MRI or PET/CT) could have better assessed soft tissue and distant spread. Given clinical suspicion of KS and inoperability of the lesion, empirical treatment with liposomal doxorubicin (20 mg/m²) was initiated. After four cycles, the lesion demonstrated marked clinical and radiologic regression. Subsequent surgical excision revealed keratinizing squamous cell carcinoma with tumor-free margins. The patient tolerated chemotherapy well, and a six-month follow-up showed no recurrence.

This case underscores the diagnostic overlap between KS and cSCC in HIV-positive patients, emphasizing the limitations of single biopsies and the need for repeated histopathology and multidisciplinary evaluation. The unexpected regression of cSCC with liposomal doxorubicin suggests potential off-label antitumor activity through DNA intercalation, topoisomerase II inhibition, and reactive oxygen species generation. While not a standard therapy for cSCC, doxorubicin may act as a bridge to definitive surgery in select high-risk or immunocompromised patients.

Clinicians should maintain high suspicion for atypical cSCC in PLHIV. Repeated histopathologic assessment and multidisciplinary management are essential. This case highlights the potential dual benefit of liposomal doxorubicin in inducing regression of non-Kaposi cutaneous malignancies, supporting further investigation into its role as a bridge to curative surgery.

## Introduction

Cutaneous malignancies are the most common cancers worldwide, and their incidence continues to rise, particularly in aging populations. Non-melanoma skin cancers (NMSC), which primarily include basal cell carcinoma (BCC) and cutaneous squamous cell carcinoma (cSCC), represent the most frequent cutaneous malignancies. While BCC is the most common overall, cSCC is the leading cause of metastasis and mortality among NMSC, accounting for significant morbidity, healthcare costs, and mortality. Approximately 2-4% of primary cSCCs metastasize, and prognosis in metastatic disease remains poor [1,2].

The global burden of cSCC is projected to increase due to cumulative ultraviolet (UV) exposure, aging populations, chronic inflammation, chronic ulcers, human papillomavirus (HPV) infection, and immunosuppression [3]. In sub-Saharan Africa, cSCC is the most common skin cancer among individuals with darker skin types, and it often presents as aggressive, multifocal lesions in persons with oculocutaneous albinism [4]. However, incidence and mortality rates of cSCC are frequently underreported, as U.S. national cancer registries do not routinely capture these data, and much of the available epidemiology originates from European and global studies [3,5].

Immunosuppressed individuals, particularly persons living with HIV (PLHIV), are at heightened risk of developing aggressive and multifocal cSCC. In these populations, clinical presentation may mimic other cutaneous malignancies such as Kaposi sarcoma (KS), complicating diagnosis and potentially delaying appropriate treatment [6]. This diagnostic overlap is clinically relevant, as KS is often treated with anthracycline-based chemotherapy such as doxorubicin, which exerts antitumor effects via DNA intercalation, topoisomerase II inhibition, and generation of reactive oxygen species. Interestingly, a few reports have observed improvement in non-Kaposi skin cancers after this medication, suggesting a possible overlap in response [7,8].

In simpler terms, people living with HIV may develop unusual or aggressive forms of skin cancer that can look very similar to Kaposi sarcoma. Recognizing this overlap is crucial, as it can delay diagnosis and affect treatment decisions.

Here, we present the case of a patient with HIV and aggressive cutaneous lesions initially suspected to represent KS, in whom histopathology confirmed cSCC. This report highlights the diagnostic challenges of distinguishing between these entities in immunocompromised hosts and underscores the potential therapeutic implications of anthracycline exposure in cSCC.

## Case presentation

**Figure 1:**
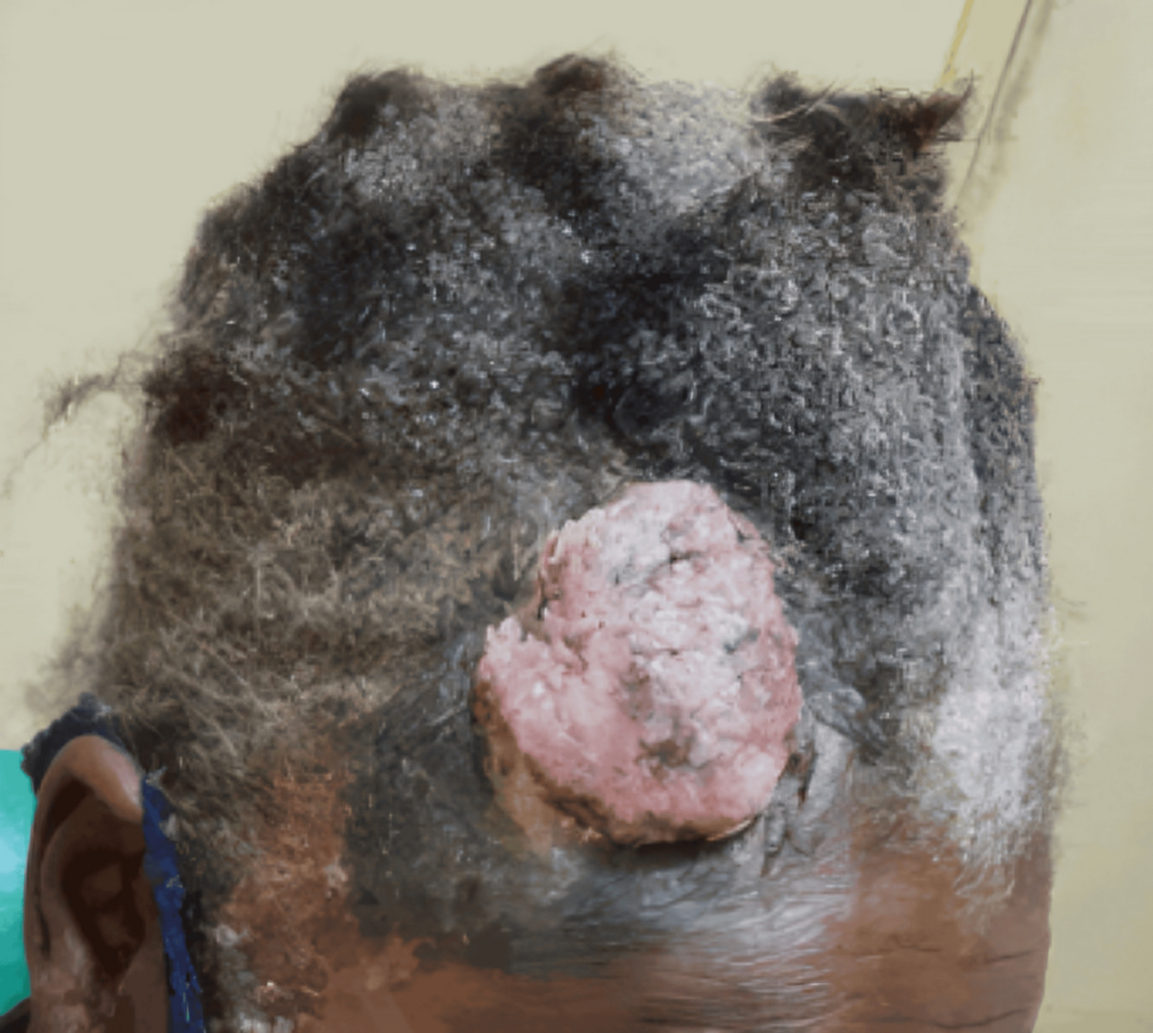
A large exophytic and ulcerated lesion on the right frontoparietal scalp. The mass is irregular, nodular, and covered with areas of crusting and necrosis. The surrounding scalp demonstrates hair thinning and local tissue distortion.

A 64-year-old female with a history of HIV infection on antiretroviral therapy (tenofovir 300 mg daily, lamivudine 300 mg daily, efavirenz 600 mg daily since 2017) presented with a progressively enlarging lesion on the right frontoparietal scalp (Figure 1). The lesion had been evolving over four months and was associated with localized pain, rapid growth, occasional bleeding, and crusting. She denied systemic symptoms such as fever, weight loss, night sweats, or malaise. Past medical history included well-controlled HIV infection, hypertension, and hyperlipidemia. There was no history of skin cancer or other malignancies, and family history was negative for skin cancer and immunodeficiency.

On physical examination, an exophytic mass measuring approximately 5 cm in diameter was noted on the right frontoparietal scalp. The lesion was firm, irregular, and tender to palpation, with purulent, fetid, yellow-green discharge. The surrounding skin showed a hyperchromic patch. Additionally, a hyperpigmented macule of about 3 cm in diameter was identified on the lower legs. The Karnofsky performance status was 90%. No lymphadenopathy or hepatosplenomegaly was observed. Laboratory evaluation demonstrated anemia, preserved leukocyte and platelet counts, and a markedly elevated erythrocyte sedimentation rate. Findings were also notable for immunosuppression reflected by a reduced CD4+ count, with the HIV viral load remaining undetectable (Table 1).

**Table 1:** Laboratory findings with reference ranges

Variable	Patient Finding	Normal Reference Range
Hemoglobin	10.5 g/dL	Male: 13.8–17.2 g/dL; Female: 12.1–15.1 g/dL
Total Leukocyte Count	4.68 × 10⁹/L	4.0–11.0 × 10⁹/L
Neutrophils (%)	49.30%	40–60%
Lymphocytes (%)	39.70%	20–40%
Platelet Count	252 × 10⁹/L	150–450 × 10⁹/L
Erythrocyte Sedimentation Rate (ESR)	172 mm/hour	Male: <15 mm/hr; Female: <20 mm/hr
CD4+ Count	171 cells/µL	500–1,600 cells/µL
HIV Viral Load	Undetectable	Undetectable in treated HIV infection

Microbiological culture of the scalp lesion exudate yielded *Staphylococcus aureus*, resistant to penicillin but sensitive to ciprofloxacin, suggesting a secondary bacterial infection complicating lesion progression. Skull radiography revealed a radiopaque lesion approximately 4 cm in diameter in the right frontoparietal region without evidence of bone infiltration or osteolytic/osteoblastic changes. Chest radiography and abdominal ultrasonography were unremarkable. Cross-sectional imaging (CT/MRI) and PET/CT were not performed but could have provided additional staging information and assessment of operability.

A punch biopsy of the scalp lesion showed inflammatory changes with atypical keratinocytes but was inconclusive. A repeat biopsy was recommended; however, it was deferred due to the lesion’s rapid enlargement, friability, and high risk of bleeding. The case was subsequently discussed at a multidisciplinary tumor board (dermatology, infectious disease, oncology, and surgery). Given the patient’s HIV-positive status, the presence of multiple hyperpigmented skin lesions, and the inoperability and bleeding tendency of the scalp mass, the team reached a consensus to initiate empirical liposomal doxorubicin therapy based on clinical suspicion of KS.

**Figure 2:**
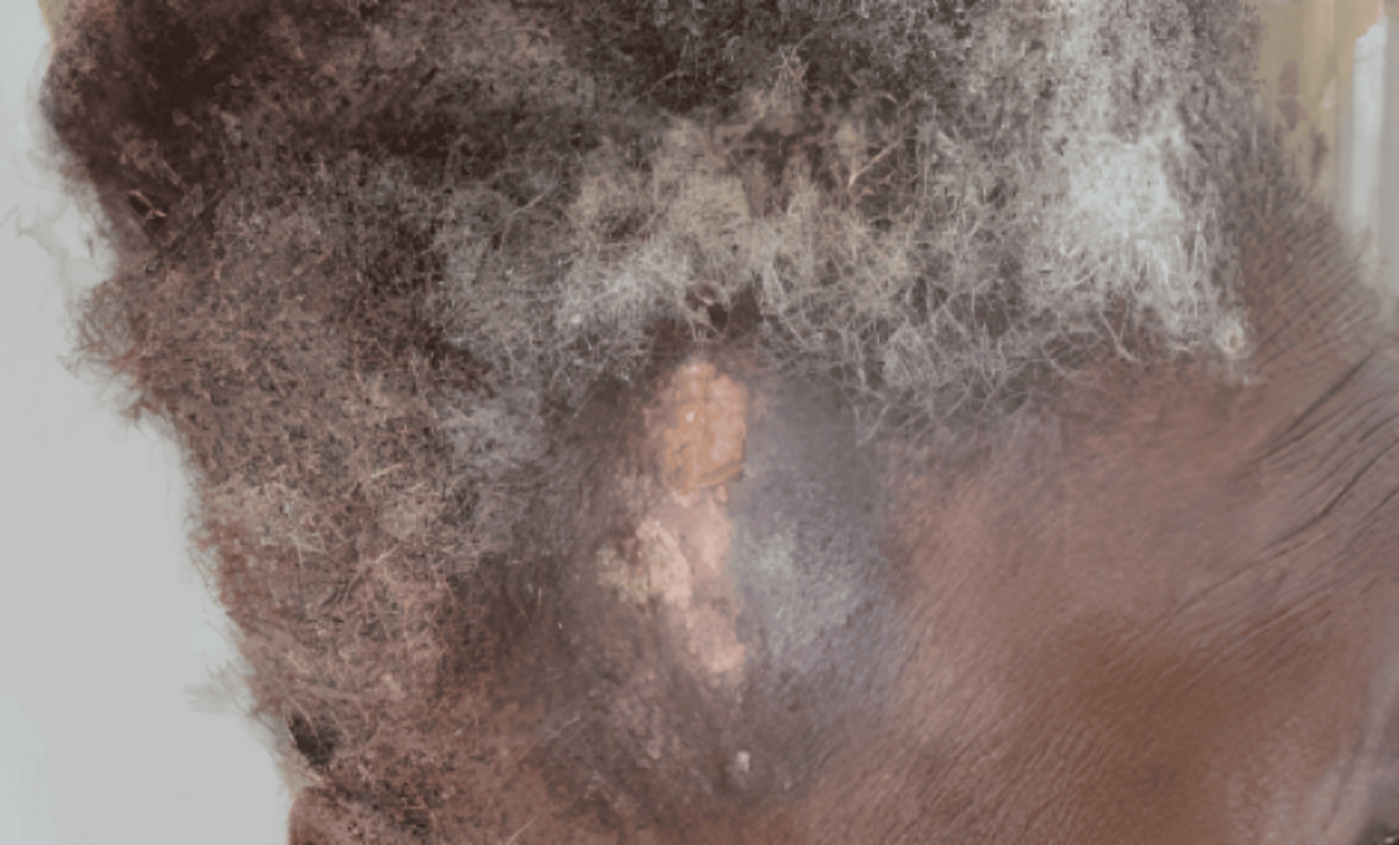
Clinical image showing the scalp region after four cycles of chemotherapy. The previously visible lesion has resolved, with healthy-appearing scalp tissue and no evidence of residual mass or ulceration.

Management included analgesia, targeted antibiotics, and systemic chemotherapy with liposomal doxorubicin at 20 mg/m² as first-line treatment for KS. After four cycles, the patient demonstrated a complete clinical and radiological response, with resolution of the scalp lesion (Figure 2), disappearance of radiographic findings, and an improved CD4+ count (345 cells/µL). She tolerated chemotherapy well, with no significant toxicity.

**Figure 3:**
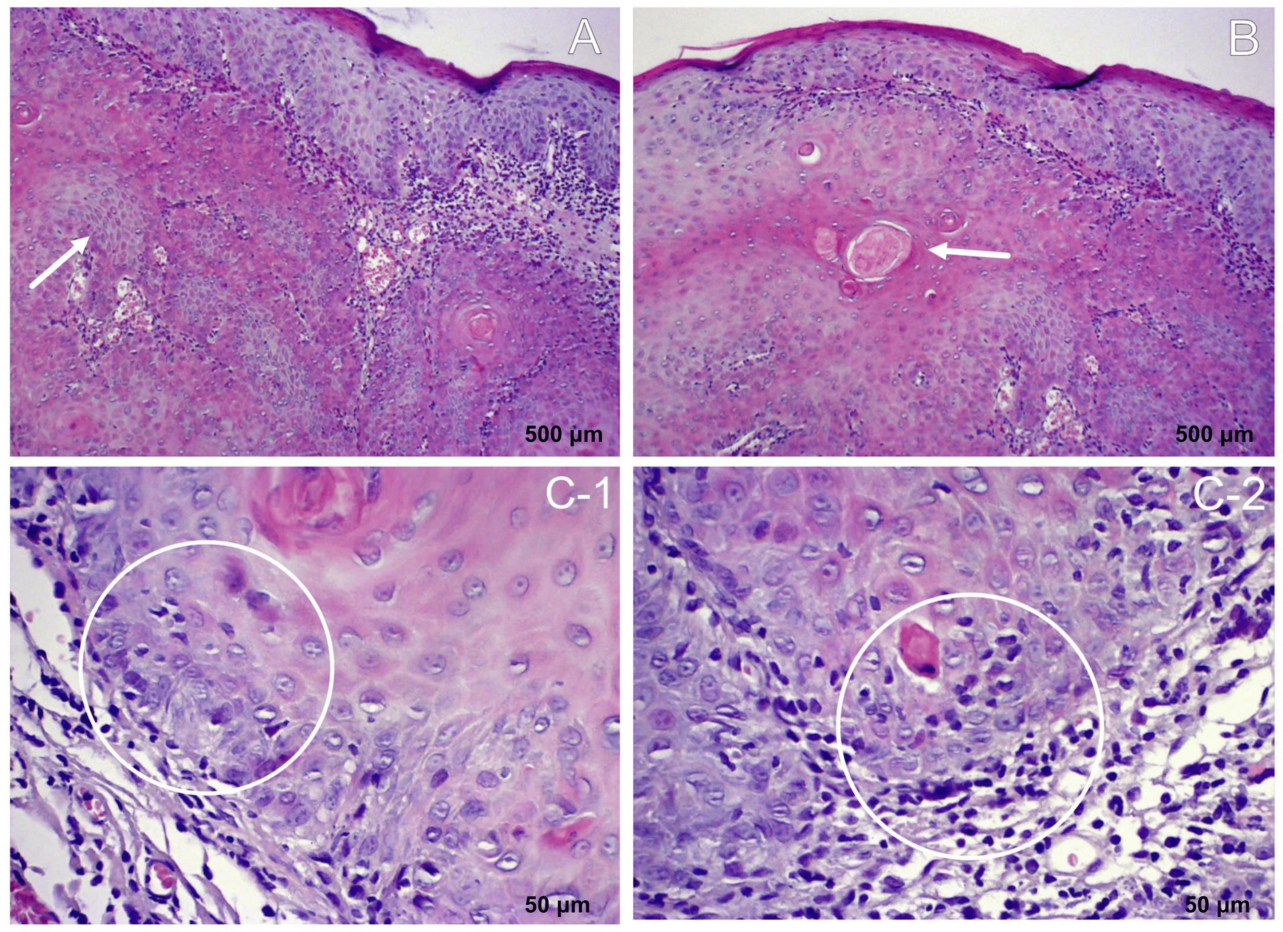
Histopathology of keratinizing squamous cell carcinoma - hematoxylin and eosin (H&E)-stained sections A: Skin specimen showing squamous cell carcinoma, composed of nests and cords of atypical squamous cells infiltrating the dermis (indicated by the white arrow). B: Keratinization and keratin pearl formation are observed, features characteristic of a well-differentiated squamous cell carcinoma, comprising approximately 40% of the specimen (keratin pearls indicated by the white arrow). C-1 and C-2: The remainder corresponds to moderately differentiated squamous cell carcinoma, characterized by greater nuclear pleomorphism and increased mitotic activity (mitotic figures indicated by the white circles).

Following chemotherapy, the patient underwent wide local excision of the residual lesion with 1-2 cm margins and local flap reconstruction. Histopathological examination unexpectedly revealed a keratinizing squamous cell carcinoma with negative margins. Immunohistochemistry confirmed keratin-positive tumor cells and the absence of HHV-8, excluding KS (Figure 3).

**Figure 4:**
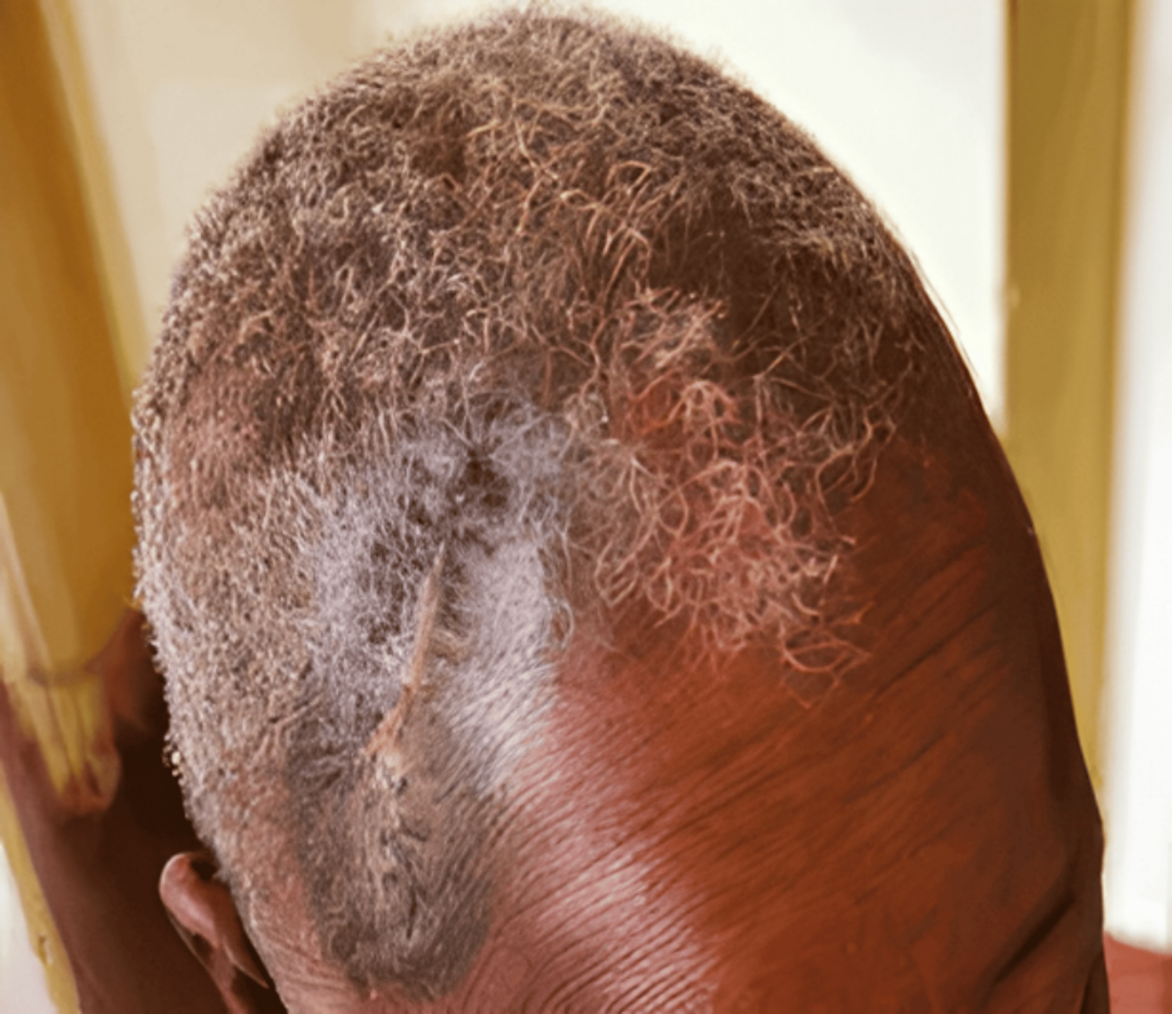
Resolution of the scalp lesion after 6 months of continued antiretroviral therapy. The previously noted cutaneous mass is no longer visible, with restoration of normal scalp contour and no evidence of new lesions or metastasis.

At six-month follow-up, there was no recurrence at the surgical site, the CD4+ count remained above 300 cells/µL, the patient continued antiretroviral therapy, and no new cutaneous lesions or metastases were observed (Figure 4).

Timeline of clinical events and treatments

Approximately 4 months before presentation: The patient noticed a small scalp lesion that progressively enlarged, becoming palpable and exophytic.

At presentation: A ~5 cm exophytic, ulcerated, bleeding scalp mass was noted in the right frontoparietal region, with localized pain, rapid growth, and occasional crusting but no systemic symptoms. Laboratory findings included hemoglobin 10.5 g/dL, ESR 172 mm/hr, CD4 171 cells/µL, and undetectable HIV viral load.

Initial workup: Wound culture was positive for *Staphylococcus aureus *(penicillin-resistant, ciprofloxacin-sensitive). Skull X-ray showed a ~4 cm radiopaque lesion without bony invasion. CT, MRI, and PET-CT were not performed.

First biopsy: A punch biopsy was performed but yielded inconclusive findings (inflammatory changes with atypical keratinocytes). Repeat biopsy was deferred due to friability and bleeding risk.

Multidisciplinary review: The case was discussed by dermatology, infectious disease, oncology, and surgery teams. Given the HIV status, hyperpigmented lesion, and high bleeding risk, an empirical trial of liposomal doxorubicin (20 mg/m²) was initiated for suspected Kaposi sarcoma (KS).

Systemic therapy: Four cycles of liposomal doxorubicin (20 mg/m²) were administered with concurrent antibiotic therapy guided by culture results and supportive care.

Post-chemotherapy assessment: After four cycles, there was marked clinical and radiologic regression of the scalp lesion, with an increase in CD4 count to 345 cells/µL. The patient tolerated treatment well without significant toxicity.

Surgical management: Wide local excision with 1-2 cm margins and local flap reconstruction was subsequently performed.

Postoperative histopathology: The definitive diagnosis was keratinizing cutaneous squamous cell carcinoma (cSCC) with negative margins. Immunohistochemistry showed cytokeratin positivity and was negative for HHV-8 (excluding KS).

Follow-up: At six months, no local recurrence, no new cutaneous lesions, and no metastatic disease were observed. The patient remained on antiretroviral therapy with regular follow-up.

## Discussion

Established evidence

Cutaneous squamous cell carcinoma represents a growing proportion of non-melanoma skin cancers worldwide, with behavior ranging from indolent lesions to aggressive tumors with metastatic potential. Immunosuppression, particularly in PLHIV, is a well-established risk factor, as chronic immune dysregulation promotes both tumorigenesis and atypical clinical presentations [9]. In this context, cSCC may closely mimic KS, especially when lesions demonstrate rapid growth, pigmentation, or ulceration [10].

In HIV-positive patients, distinguishing Kaposi sarcoma (KS) from cutaneous squamous cell carcinoma (cSCC) can be challenging due to overlapping clinical features. KS often presents as violaceous or hyperpigmented macules, plaques, or nodules, typically on the lower extremities, face, or oral mucosa, and may be multifocal. Lesions are usually non-ulcerated and slow-growing, though aggressive variants exist in advanced immunosuppression. In contrast, cSCC presents as firm, hyperkeratotic or ulcerated nodules, often on sun-exposed areas such as the scalp or face, with potential for rapid growth and local invasion. Histopathology remains essential for definitive diagnosis, particularly when clinical features overlap, and response to liposomal doxorubicin differs between the two entities.

Hypothesis and interpretation

Our case highlights several diagnostic challenges. First, the initial biopsy was inconclusive, delaying definitive diagnosis. This limitation has been previously reported, as small or superficial punch biopsies may fail to capture representative tumor architecture, particularly in ulcerated or necrotic lesions [11]. Second, the clinical context of HIV infection and hyperpigmented cutaneous lesions favored an initial impression of KS. Empirical initiation of liposomal doxorubicin, although intended for KS, unexpectedly induced tumor regression. This raises the hypothesis that anthracyclines may exert cytotoxic activity against non-Kaposi cutaneous malignancies, an observation sparsely described in the literature.

Doxorubicin, in both its conventional and pegylated liposomal formulations (pegylated liposomal doxorubicin (PLD)), remains a cornerstone in the management of KS, with randomized trials and systematic reviews supporting its role as first-line therapy [12,13,14]. Beyond KS, its potential benefit in squamous cell carcinoma (SCC) has also been explored. In head and neck SCC (HNSCC), studies have shown partial responses with PLD in untreated locally advanced cases, as well as activity in recurrent, previously treated disease with manageable toxicity. Other reports have documented objective responses in advanced cutaneous SCC using cisplatin-doxorubicin combinations, and favorable outcomes with platinum-doxorubicin-based regimens [15]. Taken together, these findings indicate that doxorubicin possesses meaningful antitumor activity in SCC, supporting its consideration as an off-label therapeutic option in selected scenarios.

However, treatment outcomes remain variable. Some studies have reported limited efficacy with etoposide-doxorubicin regimens in recurrent HNSCC, underscoring heterogeneous tumor sensitivity. Additionally, paradoxical adverse effects have been described during prolonged PLD therapy, including cases of tongue SCC and precancerous oral lesions such as leukoplakia [16]. This dual nature - potential therapeutic benefit versus risk of secondary carcinogenesis - reflects the complex interaction between anthracyclines, immunosuppression, and epithelial oncogenesis.

To our knowledge, no prior reports have documented regression of keratinizing cSCC concomitantly treated with doxorubicin for suspected KS. This underscores the importance of documenting such cases, which may provide insight into the broader antitumor potential of PLD. Importantly, this case contributes new evidence to a significant gap in the literature: while anthracyclines like doxorubicin are well established for Kaposi sarcoma, their efficacy in cSCC - particularly in HIV-associated or immunocompromised populations - remains virtually undocumented. By demonstrating objective tumor regression of keratinizing cSCC during PLD therapy, our report adds a unique clinical observation that may prompt further investigation into anthracycline responsiveness in cutaneous squamous neoplasms. This novelty lies in highlighting doxorubicin’s potential off-label cytotoxicity in epithelial tumors beyond its conventional use, bridging a previously unexplored overlap between viral oncogenesis, immune dysregulation, and chemotherapeutic response.

Clinical implications and future directions

Chemotherapy-induced regression in our patient allowed subsequent surgical excision with curative margins, demonstrating how systemic therapy can serve as a bridge to definitive local management in otherwise inoperable cases. Still, histopathology remains the gold standard for differentiating cSCC from KS, both of which may coexist in HIV-positive patients. Notably, while published evidence of doxorubicin efficacy in cutaneous SCC is lacking, an ongoing phase I/II clinical trial (NCT05377905) is currently evaluating the safety and local tumor response of a microneedle array delivering doxorubicin directly into cSCC lesions, highlighting the emerging interest in this therapeutic approach [15, 17].

This case adds to the limited literature exploring the intersection of HIV-related immunosuppression, diagnostic uncertainty, and therapeutic response in cutaneous malignancies. It reinforces the need for repeated biopsies when results are inconclusive, multidisciplinary evaluation, and flexibility in therapeutic planning for high-risk immunocompromised patients. 

Future research should further explore whether PLD has a broader role in cSCC management, particularly in populations where standard treatments may be limited or contraindicated.

Limitations

This report has several limitations. It describes a single-patient case, which limits the generalizability of the findings. Additionally, advanced imaging techniques such as MRI or PET/CT were not performed, which could have provided more precise staging and assessment of disease extent. These factors should be considered when interpreting the observed therapeutic response.

## Conclusions

This case illustrates the diagnostic and therapeutic challenges of cutaneous malignancies in HIV-positive patients, where clinical overlap between KS and cutaneous cSCC, combined with inconclusive biopsies, may delay appropriate treatment. In our patient, empirical liposomal doxorubicin - initiated for suspected KS - induced marked regression of cSCC, enabling definitive surgical excision with curative margins.

These findings suggest a possible off-label role for liposomal doxorubicin as a bridge to surgery in selected high-risk or immunocompromised patients with unresectable lesions. By documenting this unexpected response, the case expands current knowledge on anthracycline activity in non-Kaposi cutaneous malignancies and highlights the need for further clinical studies assessing PLD’s therapeutic potential in cSCC.

Clinicians should maintain vigilance for atypical cSCC presentations in people living with HIV, pursue repeated biopsies when necessary, and engage in early multidisciplinary management to optimize outcomes.
